# Job Crafting Among American Workers with Disabilities

**DOI:** 10.1007/s10926-020-09889-9

**Published:** 2020-04-13

**Authors:** Debra L. Brucker, Vidya Sundar

**Affiliations:** 1 University of New Hampshire, Institute on Disability, 10 West Edge Drive, Suite 101, Durham, NH 03824 USA; 2 University of New Hampshire, Occupational Therapy Department, 115 Hewitt Hall, 4 Library Way, Durham, NH 03824 USA

**Keywords:** Job crafting, Disability, Work, Employment

## Abstract

*Purpose* Job crafting is an informal, employee-initiated approach to job re-design that has not been tested among people with disabilities, thus far. The purpose of this study is to examine crafting behaviors of workers with disabilities and individual factors associated with crafting behaviors. *Methods* We conducted a survey of employees with disabilities who were 18–64 years old and had at least 1 year of work experience. Bivariate and multivariate methods were used to: (1) compare the use of job crafting behaviors between our sample and published results from a sample of the general population; (2) identify individual characteristics associated with job crafting for workers with disabilities. *Results* Persons with disabilities engage in job crafting behaviors, albeit at lower levels than that reported in a broader sample (Int J Wellbeing, https://doi.org/10.5502/ijw.v3i2.1, 2013). Education, and disability type (visual and mobility impairment) were associated with certain types of crafting behaviors. *Conclusions* As job crafting can be associated with higher levels of engagement and career growth among persons without disabilities, findings from this research can be utilized to design programs and policies that support the career goals of people with disabilities beyond labor force participation.

## Introduction

In the United States (U.S.), rates of employment are substantially lower for working-age persons with disabilities than for others without disabilities. In 2016, 36% of the approximately 20 million working-age persons with disabilities were employed, compared to 77% of those without disabilities [1]. While much research has attempted to explain this gap in employment rates [2, 3], less attention has been paid to the actual experience of workers with disabilities in the workplace. Understanding how workers with disabilities navigate the demands of the workplace can provide information that can be used by individuals with disabilities as well as the service providers that assist them with employment-related activities to develop effective employment retention strategies. Much of the current research and literature on employment for individuals with disabilities focuses on accommodation strategies associated with requesting and acquiring reasonable accommodations. However, there is emerging evidence suggesting that reasonable accommodation requests carry a certain amount of risk in the workplace [4–7]. Most employees with disabilities function without the benefit of outside services and supports, instead relying on their individual ability to manage workplace tasks and relationships. Federal/state vocational rehabilitation (VR) programs, for example, provided career services to 737,077 clients in federal fiscal year 2017, including 64,420 youth age 18 and younger [8]. Many of the services provided by VR focus on career readiness and thus likely serve a smaller number of individuals who are actively in the workforce.

Job crafting is an employee-initiated endeavor to actively change perceived and tangible aspects of one’s job. Workers with disabilities may be engaging in job crafting behaviors by negotiating and modifying job tasks, perceptions of their job roles and tasks, and social interactions in the workplace to suit their unique needs, skills and values. Among the general population, job crafting has been found to be effective in enabling occupational performance [9]. We hypothesize that some individual workers with disabilities participate in job crafting behaviors, without the benefit of coaching.

## Literature Review

Job crafting is the process by which employees take active steps in defining and designing their own job experience in a personally meaningful way [9]. A person’s work life and career can ultimately be deconstructed to the day-to-day job tasks they perform, the people they interact with, and the value and meaning they attach to their jobs. While the specific job tasks employees engage in are primarily determined by job descriptions, most employees have some latitude in determining how they perform job tasks. For example, a restaurant manager who is responsible for ordering supplies and stock may choose to do the same online versus via a phone call, depending on his/her personal preference, thus crafting how he/she performs the job.

Job crafting is a ‘bottom-up’ approach to job redesign [10, 11] and occurs in three primary areas: (1) Task crafting refers to changes in job tasks and how they are performed. Task crafting occurs when employees take on additional responsibilities, emphasizing certain job tasks or redesigning job tasks [12]. For people with disabilities, task crafting can include informal alternate ways of performing a job task, the use of assistive technology, reasonable accommodations, or job redesign. (2) Relational crafting refers to changing the extent or nature of one’s interactions with people within and outside the organization. Relational crafting occurs when workers build new relationships, reframe existing relationships, and adapt relationships. Relational crafting can also be embedded within task crafting, wherein social interactions are molded within the context of a task, thereby altering the way a task is performed. (3) Cognitive crafting involves changing perceptions about one’s job or job tasks to enhance meaningfulness. This is a mental or cognitive type of job crafting since it does not involve making any physical or social changes but rather involves reshaping of one’s own thoughts and perceptions about one’s job. Cognitive crafting can take the form of expanding perceptions, focusing perceptions, or linking perceptions where people make connections between different aspects of their job tasks to create a meaningful schema.

Job crafting has been found to be positively associated with levels of work engagement [13–15]. Internal job crafting, defined as cognitive actions within a person, is negatively associated with job satisfaction and is positively associated with structural job crafting [11]. Internal job crafting is positively related to job burnout in that those who engage in internal job crafting are more likely to experience heightened emotional exhaustion and depersonalization. Structural job crafting, defined as behavioral interactions within the work environment, is negatively related to burnout dimensions (emotional exhaustion, depersonalization, lack of accomplishment) [11].

Prior research among the general population has suggested that high levels of self-efficacy and proactive personality traits [15, 16] are positively associated with job crafting behaviors. Self-efficacy is important not only for career success but also for long-term career trajectories [17]. Social cognitive theory [18–20] posits that all individuals strive for a sense of agency or control over their lives and that this agency results from a dynamic process among behavioral, environmental and personal interactions. This has relevance for job crafting as individuals may engage in job crafting behaviors to foster their sense of agency in the workplace. Job crafting can also be thought of as embodying certain aspects of positive psychology theory. As positive psychology theory suggests that individuals derive different meanings from work based on an interplay of thoughts, feelings and behaviors, elements of job crafting reflect this viewpoint [21].

A recent meta-analysis of job crafting research conducted on the general population noted two types of intrinsic motives that might influence an individual’s motivation to undertake job crafting: proactive and reactive motives. “Proactive motives refer to employees wanting to initiate job crafting to reach desirable goals, while reactive motives are related to the need to cope with adversity” [22]. Lazazzara and colleagues [22] identified three different types of job crafting dimensions: approach crafting in which employees add extra tasks or reframe work roles, avoidance crafting where workers reduce workplace roles and limit social ties, and, crafting in other domains. All can occur with each type of job crafting (cognitive, relational and task). A consideration of the mix of motives as well as the dimensions listed above may be particularly relevant when considering how workers with disabilities craft their jobs.

Demerouti and Peeters [23] showed that crafting aimed at minimizing job demands may be a protective or reactive mechanism to address one’s health or emotional exhaustion. Many individuals with disabilities strive to work, overcome barriers in the workplace, and retain their jobs [3]. They do so by actively managing their careers, advocating for themselves, and adapting to work roles for successful integration in the workplace [24]. According to data from the 2015 Kessler Foundation National Employment and Disability Survey (KFNEDS), 16% of workers with disabilities have faced negative attitudes on the part of supervisors and 41% of those workers have stated that they have overcome those barriers. Similarly, 16% of workers with disabilities have faced negative attitudes on the part of co-workers and 55% of those workers stated they have overcome those barriers [3]. Some of the workers who were successful in overcoming these barriers may have used idiosyncratic deals [25] or innate job crafting skills.

The most frequently used types of workplace accommodation are flexible schedules and modified job duties [3, 25]. Acquiring these accommodations can happen through a formal request, where the employee discloses their disability and requests a reasonable accommodation under the ADA or informally where employees make deals to re-arrange their schedule with their employer or co-workers [25, 26]. Dong and colleagues [26] showed that accommodations requests through informal channels were more popular than formal requests. While it is helpful to understand, for example, the 28% of workers with disabilities have a flexible schedule and 14% of workers with disabilities have modified job duties, more information is needed about how accommodations are implemented for workers with disabilities and whether job crafting skills were used to request those accommodations [3]. To date, however, evidence demonstrating the use of job crafting by workers with disabilities has been minimal.

Markel and Barclay [27] conducted interviews with 17 workers with disabilities who were professionally employed and had at least an undergraduate degree to determine how workers with disabilities navigate the employment and accommodation process. They found that the timing of disability onset and the presence of a key support person were important factors associated with the successful navigation of professional needs. The study participants did not rely on the human resources department of their employer to receive workplace accommodations, and instead, individually crafted their jobs to develop careers. Decisional control, self-advocacy, and persistence have been found to be protective of employment for people dealing with pain and other chronic conditions [28, 29]. Tait [30] recommends that those at risk of job loss due to pain would benefit from psychosocial interventions, including job crafting, that can assist individuals in effectively managing limited personal resources when faced with chronic pain.

The purpose of this paper is to extend research on job crafting to the population with disabilities, investigating whether workers with disabilities informally participate in job crafting behaviors and if so, what individual characteristics might be associated with job crafting behaviors. Our specific research questions are:

1. Are workers with disabilities less likely to participate in job crafting than others?
2. Among workers with disabilities, what individual characteristics are associated with higher levels of job crafting?

## Methods

### Data

We use quantitative data from a survey we conducted in 2016. Participants were members of a voluntary panel maintained by Qualtrics [31], an online survey software company, and its partner organizations. Respondents were recruited by Qualtrics and its partner organizations using a variety of methods, including web intercept, targeted email lists, panel member referral, and social media. Incentives for respondents included cash payments, free downloads, and/or membership points; all incentives were decided and allocated by Qualtrics and its partners. Informed consent to participate was obtained in accordance with requirements of the University of New Hampshire Institutional Review Board, and respondents were verified by Qualtrics through a double opt-in process.

Respondents were included in the survey if they were adults between the ages of 18 and 64 with one or more disabilities or chronic health conditions. Electronic consent to participate, in accordance with protocols of the University of New Hampshire Institutional Review Board, was granted by 11,045 individuals. Of those, 4,259 were precluded from taking the survey because they indicated no disability or health condition, and 3,181 were not admitted to the survey for being over age 64. Another 583 were dropped for inattentive responding, which means that respondents incorrectly answered at least one Likert-type item designed to assess whether the questions were being thoroughly read. The median time to complete the survey was 13 minutes. As there were several different tracks through the survey, and some were very short, no participants were excluded based on time to complete the survey. Instead, responses with very short duration times were reviewed individually to verify that they belonged to the shortest survey track. This resulted in no further exclusions. The analytic sample for this study comprised the subset of the remaining 3,022 participants who (a) were between the ages of 18 and 64, (b) were currently employed, and (c) responded to all the job crafting questions. This resulted in a final analytic sample of 753 workers with disabilities.

### Measures

Task, relational and cognitive forms of job crafting were measured using questions developed and validated by Slemp and Vella-Brodrick [32] in their 15-item Job Crafting Questionnaire (JCQ). The JCQ has high reliability for the entire scale (Cronbach’s alpha = 0.91) as well as for each sub-scale: task crafting Cronbach’s alpha = 0.87; relational crafting Cronbach’s alpha = 0.83; cognitive crafting Cronbach’s alpha = 0.89) [32]. For each question, respondents were asked how often they engaged in certain crafting tasks. Responses could range from 1 (“Not at all”) to 6 (“Very often”). The variables were treated as continuous variables for some of the analysis described below and were also recoded into binary variables, where individuals with scores at or above the median were coded as one and individuals with scores below the median were coded as zero, for additional analyses. This recoding addresses the skewed nature of the data.

Demographic characteristics were measured as well. Age was measured at an interval level. Sex was measured as male or female. Race was measured as Caucasian or not. Educational attainment was measured as high school or less, some college or technical school, and college graduate or more education. Primary type of disability was measured as cognitive, hearing, mobility, vision, or other, based on respondent self-report of their primary disability. The category of cognitive limitations included developmental disabilities such as Down’s Syndrome or autism as well as emotional, psychological, or mental health disabilities such as depression, anxiety and other conditions. Mobility limitations identified those persons who stated that they had serious difficulty walking or climbing stairs, walking a quarter of a mile, or doing physical activities such as lifting, carrying, bending or manipulating small objects. Disability onset was measured as adult onset (age 18 or older) or not, based on respondent self-report.

### Analytical Approach

Descriptive statistics for the sample characteristics as well as the job crafting questions are first provided. Our sample data was then weighted to mirror the sample characteristics of the data used by Slemp and Vella-Brodrick [32], adjusting for sex and full-time employment status. To address our first research question, the JCQ items were treated as continuous variables to compare the mean values to those reported by Slemp and Vella-Brodrick [32], using two independent sample t-tests with unequal variances assumed.

To address our second research question, the data was first re-weighted to match the composition of the working-age population based on age, educational attainment, sex, and race data from the U.S. Census Bureau’s American Community Survey (ACS) [33] We then ran a series of logistic regressions. The dependent variable for each regression was participation in a job crafting behavior. The following individual characteristics were included as predictors on the full sample: Age, disability type, educational attainment, sex, and race. For the subset of the sample that had data for disability onset (72.9% of the sample), disability onset was added as an additional covariate.

## Results

Table 1 presents unweighted and weighted descriptive statistics of our data. Data weighted using the ACS are reviewed here. The mean age of workers with disabilities was 40.82 (Std. Dev. 12.922). Slightly more females (53.7%) than males (46.3%) were represented among workers with disabilities. Sixty-one percent of workers with disabilities were Caucasian. Equal proportions of workers with disabilities had high school or less and college or more educations (38.3%). Cognitive and mobility limitations were most often reported as the primary disability (50.0 and 35.4%, respectively).

**Table 1:** Demographic characteristics

Sample characteristics of workers with disabilities, ages 19–66	Unweighted	Weighted (SVB)	Weighted (ACS)
Age			
Mean	53.310	40.820	40.820
SD	9.914	12.925	12.922

	%	%	%
Sex			
Male	32.8	34.9	46.3
Female	67.2	65.1	53.7
Race			
Caucasian	88.1	93.9	60.9
Other	11.9	6.1	39.1
Education			
High school or less	13.3	12.3	38.3
Some college or technical school	31.8	31.6	23.4
College graduate or more	54.8	56.1	38.3
Primary disability			
Cognitive	42.1	42.2	50.0
Hearing	11.5	12.3	5.4
Mobility	35.5	34.8	35.4
Vision	7.1	7.2	7.5
Other	3.7	3.4	1.8
Disability onset			
Adult onset	72.9	69.0	72.9
Not	27.1	31.0	27.1

Table 2 presents descriptive statistics of the job crafting questions (mean, standard deviation) and compares those results to results published by Slemp and Vella-Brodrick [32], using two independent sample t-tests with unequal variances assumed. In most cases, the mean scores found for our sample of workers with disabilities were significantly lower than the mean scores reported by Slemp and Vella-Brodrick [32]. Two exceptions included the mean scores for reminding yourself about the importance of work for the broader community and thinking about the ways in which your work positively impacts your life. Mean scores between workers with disabilities and the population studied by Slemp and Vella-Brodrick [32] did not differ significantly for these two types of cognitive crafting behaviors.

**Table 2:** Comparison of mean scores

How often do you engage in the following; (1 = Not at all … 6 = Very often)	Brucker and Sundar (2016)				Slemp and Vella-Brodrick (2013)				*t*	*df*	*p*
	Mean		SD		Mean		SD				
Task crafting											
Introduce new approaches to improve your work	3.13		1.44		3.94		1.48		− 6.150	208	< 0.001
Change the scope or types of tasks you complete at work	2.73		1.38		3.54		1.47		− 6.226	214	< 0.001
Introduce new work tasks that you think better suit your skills or interests	2.65		1.44		3.42		1.47		− 5.882	213	< 0.001
Choose to take on additional tasks at work	3.33		1.52		4.12		1.34		− 6.444	205	< 0.001
Give preference to work tasks that suit your skills or interests	3.31		1.48		4.09		1.39		− 6.210	177	< 0.001
Task crafting total	3.03				3.82						
Cognitive crafting											
Think about how your job gives your life purpose	3.18		1.63		3.69		1.46		− 3.832	218	0.000
Remind yourself about the significance your work has for the success of the organization	3.49		1.58		3.48		1.41		0.078	251	0.938
Remind yourself of the importance of your work for the broader community	3.23		1.61		3.45		1.53		− 1.595	242	0.112
Think about the ways in which your work positively impacts your life	3.38		1.52		3.66		1.43		− 2.167	238	0.031
Reflect on the role your job has for your overall well-being	3.42		1.50		3.96		1.33		− 4.444	211	< 0.001
Cognitive crafting total	3.34				3.65						
Relational crafting											
Make an effort to get to know people well at work	3.65		1.51		4.24		1.24		− 5.122	278	< 0.001
Organize or attend work related social functions	2.60		1.56		3.39		1.56		− 5.667	224	< 0.001
Organize special events in the workplace	2.33		1.51		3.16		1.61		− 5.825	198	< 0.001
Choose to mentor new employees (officially or unofficially)	2.82		1.61		3.48		1.51		− 4.835	191	< 0.001
Make friends with people at work who have similar skills or interests	3.36		1.58		4.09		1.33		− 5.945	211	< 0.001
Relational crafting total	2.95				3.67						

*p < 0.05, ***p < 0.001

Table 3 shows results from the logistic regressions on the full sample, predicting the likelihood of participating in each job crafting behavior. Age, sex, and race were not significantly associated with job crafting behavior. Education was associated with job crafting behaviors, even when controlling for age, sex, race and type of disability. Compared to those with high school degrees or less education, workers with disabilities who had some college education had significantly higher odds of engaging in one task crafting behavior (giving preference to work tasks that suit skills or interests, OR 2.713, *p* = 0.025) and one relational crafting behavior (making friends with people at work who have similar skills and interests, OR 2.680, *p* = 0.031). Workers with bachelor’s degrees or more had significantly increased odds of participating in all task, cognitive, and relational types of job crafting, with odds ratios ranging from 2.947 to 4.855.

**Table 3:** Adjusted odds ratios of job crafting behaviors, full sample

	Age	Caucasian	Male	Tech/some college	College +	Cognitive	Mobility	Hearing	Vision	Constant
	Exp(B)	Exp(B)	Exp(B)	Exp(B)	Exp(B)	Exp(B)	Exp(B)	Exp(B)	Exp(B)	Exp(B)
Task crafting										
Introduce new approaches	0.996	1.226	0.940	2.322	3.860***	0.412	0.125***	0.489	0.17**	0.685
Change scope/types of tasks	0.993	0.968	1.530	1.642	3.760***	0.942	0.472	1.207	0.404	0.240
Introduce new tasks to suit skills/interests	0.999	1.715	1.339	1.681	2.947*	0.666	0.335	1.034	0.465	0.163
Choose additional tasks	0.999	1.396	0.887	2.146	3.706***	0.515	0.134***	0.575	0.199*	0.472
Preference to tasks that suit skills/interests	1.001	1.389	0.995	2.713*	4.855***	0.715	0.197**	0.751	0.2595*	0.265
Cognitive crafting										
Think about how job gives purpose	1.000	1.328	0.759	1.947	3.114**	0.647	0.216**	0.763	0.310	0.362
Remind yourself about significance of work	1.010	1.147	0.996	1.801	3.466**	0.467	0.256*	0.648	0.270	0.190
Remind yourself of importance of work	1.002	1.077	1.184	2.074	4.604***	1.844	0.790	2.255	0.807	0.088*
Think about how work positively impacts life	1.006	1.993	0.777	2.067	3.174**	0.524	0.214**	0.677	0.287	0.238
Reflect on role of job for well-being	1.005	0.966	0.698	2.231	3.216*	0.683	0.177**	0.808	0.379	0.266
Relational crafting										
Get to know people well	1.009	1.116	0.923	2.197	3.344*	0.500	0.144***	0.680	0.295	0.227
Organize or attend social functions	1.000	1.806	1.157	1.712	3.586**	0.717	0.241*	0.846	0.348	0.217
Organize special events	0.985	1.638	1.455	1.756	3.881**	0.900	0.485	1.035	0.490	0.238
Mentor new employees	1.002	1.700	1.595	1.440	3.412**	0.741	0.526	1.137	0.422	0.1088*
Make friends with people	1.006	1.956	1.230	2.6796*	3.922**	1.227	0.411	1.560	0.587	0.074**

*p < 0.05; **p < 0.01; ***p < 0.001

Some differences were noted by disability type. Compared to those with other types of disabilities, workers with mobility limitations had significantly lower odds of participating in three task crafting behaviors (introducing new approaches to improve work, OR 0.125, *p* < 0.001; choosing to take on additional tasks at work, OR 0.134, *p* < 0.001; and, giving preference to work tasks that suit skills and interests, OR 0.197, *p* = 0.001); three cognitive crafting behaviors (thinking about how a job gives your life a purpose, OR 0.216, *p* = 0.007; thinking about the ways in which work positively impacts your life, OR 0.214, *p* = 0.004; and, reflecting on the role your job has for your overall well-being, OR 0.177, *p* = 0.008), and one relational crafting behavior (making an effort to get to know people well at work, OR 0.144, *p* = 0.001). Workers with visual limitations had reduced odds of introducing new approaches to improve their work (OR 0.170, *p* = 0.008), choosing to take on additional tasks at work (OR 0.260, *p* = 0.040), and, giving preference to work tasks that suit their skills or interests (OR 0.260, *p* = 0.040).

Table 4 presents the results from the regressions that include the disability onset variable. Our focal variable for this smaller sample was disability onset, a variable that was significantly associated with only one type of job crafting. Workers with adult onset of disability had slightly increased odds (OR 1.002, *p* = 0.010) of thinking about how their jobs gave their lives purpose. Age, sex and race were not associated with the odds of engaging in job task, cognitive or relational crafting. The association of educational attainment and crafting was muted, as those with bachelor’s or more had increased odds of engaging in only one type of task crafting and one type of cognitive crafting. Mobility limitations were associated with reduced odds of participating in a range (but not all) types of crafting.

**Table 4:** Adjusted odds ratios of job crafting behaviors, sample subset that included disability onset variable

	Age	Caucasian	Male	Adult onset	Tech/some college	College +	Cognitive	Mobility	Hearing	Vision	Constant
	Exp (*B*)	Exp (*B*)	Exp (*B*)	Exp (*B*)	Exp (*B*)	Exp (*B*)	Exp (*B*)	Exp (*B*)	Exp (*B*)	Exp (*B*)	Exp (*B*)
Task crafting											
Introduce new approaches	0.991	0.544	1.155	1.983	1.661	2.196	0.603	0.128**	0.380	0.0567*	1.424
Change scope/types of tasks	0.979	0.481	2.161	1.395	1.295	2.399	1.149	1.128	0.989	0.099	0.737
Introduce new tasks to suit skills/interests	1.005	0.995	1.224	1.594	1.311	1.501	0.853	0.369	1.049	0.267	0.229
Choose additional tasks	0.995	0.552	0.979	1.830	1.456	2.074	0.721	0.163**	0.448	0.063*	1.206
Preference to tasks that suit skills/interests	0.993	0.685	1.437	1.638	2.452	3.288*	1.020	0.222*	0.581	0.089*	0.517
Cognitive crafting											
Think about how job gives purpose	0.993	0.622	0.923	2.648**	1.218	1.823	1.025	0.209	0.767	0.110	0.586
Remind yourself about significance of work	1.010	0.424	0.943	0.525	1.203	2.445	0.429	0.551	0.245	0.073*	1.124
Remind yourself of importance of work	0.987	0.319*	1.395	1.156	1.231	3.195*	2.218	1.665	1.224	0.145	0.557
Think about how work positively impacts life	1.010	0.908	0.664	1.534	1.403	1.625	0.702	0.178**	0.516	0.129*	0.505
Reflect on role of job for well-being	0.990	0.278**	0.909	1.586	1.637	2.252	0.963	0.174*	0.554	0.056*	1.098
Relational crafting											
Get to know people well	1.005	0.607	1.337	1.982	1.725	2.267	0.705	0.177*	0.717	0.106	0.287
Organize or attend social functions	0.996	0.953	1.668	2.025	1.447	2.299	1.191	0.230*	0.958	0.128	0.272
Organize special events	0.975	0.585	1.970	1.184	1.537	2.688	1.236	0.944	0.848	0.125	0.836
Mentor new employees	1.005	0.812	1.567	0.881	1.049	2.382	0.831	0.966	0.965	0.179	0.276
Make friends with people	0.999	1.159	1.965	2.034	2.039	2.637	2.080	0.491	2.021	0.266	0.095*

*p < 0.05; **p < 0.01; ***p < 0.000

## Discussion

Workers with disabilities do participate in job crafting behaviors, but not at the level found when studying a broader population [32]. This finding addresses our first research question. Participants in our sample scored the lowest in the relational crafting scale, followed by the task and cognitive crafting domains. In contrast, participants in the broader sample scored consistently in all three domains of crafting. In general, employees with disabilities seemed less likely as a group to engage socially with co-workers by attending work related social events or mentoring new employees (officially or unofficially). Our findings are consistent with previous research that suggests individuals with disabilities experience limited social interactions in the workplace [34, 35]. Cognitive crafting is the process of reflecting on the meaning and significance attached to work. On average, our sample scored the highest in this domain, suggesting that employees with disabilities perceive a heightened sense of purpose and meaning through their jobs. Cognitive crafting is invariably linked to task and relational crafting because workers derive meaning from engaging in fulfilling tasks, utilizing their skills, and building lasting relationships with co-workers [36]. Our findings suggest that persons with disabilities perceive a high level of personal significance despite not being able to craft their work tasks and social relationships at work. As job crafting has been found to be associated with better attachment to employment [14, 15], the development of strategies to increase the adoption of job crafting behaviors among persons with disabilities is warranted and may help to increase work engagement and job retention among employees with disabilities.

The results presented here demonstrate that persons with disabilities are less likely to participate in task, cognitive or relational crafting strategies than the population as a whole, suggesting that an approach to increase the use of job crafting for this population would need to cover all types of job crafting while also being attuned to the additional workplace challenges and barriers that persons with disabilities may face. The biggest difference between our sample and Slemp and Vella-Brodrick’s sample was noted in the items “choose to take on additional tasks at work”, “change the scope or types of tasks you complete at work”, and, “give preference to work tasks that suit your skills or interests.” Our results are consistent with previous studies which have noted that individuals with disabilities or health issues engage in crafting behaviors that are protective of their health and function. At the same time, job role expansion by taking on additional responsibilities and altering the scope of work is the steppingstone to career growth and development [37–39]. Our study suggests that employees with disabilities may not be taking advantage of these opportunities.

In general, the smallest difference between people with and without disabilities was noted in the cognitive crafting domain. There were no significant differences in the items “remind yourself of the importance of your work for the broader community” and “remind yourself about the significance your work has for the success of the organization” between our sample and Slemp and Vella-Brodrick’s [32] sample. It is probable that employees with disabilities consider their jobs just as important as those in the broader sample, despite experiencing fewer opportunities to craft their jobs tasks. Interventions and tools for job crafting among the general population are currently being used, including one-day training interventions [40] and hard copy and online workbooks [12], however, these tools likely require modification to address the unique needs of workers with disabilities.

In terms of our second research question, our first set of regressions identified two individual characteristics of workers with disabilities that increase the odds of participating in specific job crafting activities. First, higher levels of educational attainment were associated with higher participation in job crafting behaviors for workers with disabilities. This finding mirrors prior research conducted among the general population that has found that persons with higher levels of education are more likely to craft their jobs [41] and that persons with disabilities who have higher levels of education are more likely to work in higher quality jobs which offer greater autonomy [42]. While it is possible that those with higher levels of educational attainment were working in jobs which afforded greater flexibility to craft tasks and to participate in relational crafting, our findings emphasize that workers with disabilities who are highly educated more often engage in all types of crafting, including cognitive crafting. Highly educated workers with disabilities may in fact be more comfortable changing their own perceptions of their jobs rather than attempting to change more tangible facets of their workplace experiences. It remains unclear if highly educated workers with disabilities downgrade or lower their expectations to meet the demands of their jobs. Those working in low-wage hourly positions as a result of having lower levels of education might not be afforded the opportunity to craft their job tasks or to take time to establish workplace relationships but may have more latitude in engaging in cognitive crafting if given access to the appropriate tools or training.

Next, we noted minor differences by type of disability. Compared to those with other types of disabilities, workers with mobility limitations had reduced odds of participating in most job crafting behaviors. In addition, we noted some disparities in job crafting among those with visual impairments. To increase job crafting among these populations, future research should focus specifically on these sub-populations, collecting qualitative and quantitative data that can explain these disparities and identify areas of possible intervention or training. For the subset of regressions that include disability onset as a covariate, those with adult onset of disability had significantly increased odds of thinking about how their jobs gave their life purpose (OR 1.002, *p* < 0.010). No other differences were noted. Our finding that disability onset is, for the most part, not significantly associated with job crafting behaviors is counter to that of Markel and Barclay [27], although their study included a small sample (N = 17) of college-educated workers. The population of employees with disabilities is heterogeneous, however, not just in terms of sociodemographic characteristics and disability type, but also in the timing of disability onset. While our results did not isolate disability onset as a predictor of most job crafting behaviors among workers with disabilities, other research can examine this possible relationship in further detail. Workers who have congenital or early onset of disability may have gradually learned skills throughout the course of their lives that can easily translate into job crafting behaviors in the workplace. Workers who acquire disability at later ages while already employed may therefore require more intensive and targeted guidance about how to craft their jobs. Future research that examines the intersection of sociodemographic characteristics with the likelihood of engaging in job crafting behaviors should be sure to include disability type, severity of disability and age of disability onset as key characteristics so that additional information can be gained about the possible interactions among these factors with job crafting.

Several limitations of this analysis must be noted. First, our sample of workers with disabilities did not include a comparable sample of workers without disabilities. While we attempted to address this concern by comparing our results to research conducted by others, having our own sample would have improved our ability to state with confidence what the association of disability is with job crafting behaviors. Similarly, our data were not stratified by severity of disability. Being able to do so would improve our ability to develop targeted findings and recommendations about how to increase job crafting behaviors among workers with different levels of severity. Future research in this area should be sure to include adequate samples of those with and without different types of disabilities and among persons with different levels of disability severity so that more specific recommendations can be made.

Second, our sample was unique in its over-representation of those with cognitive limitations in in the workplace. Future research which uses a more representative sample would afford opportunities to further explore differences in job crafting among persons with different types of primary limitations.

Third, omitted variables bias is a concern. The survey we conducted did not include enough detail about employment situations (hours of work, type of work, choice to work full v. part time, rate of pay, organizational structure, etc.) to be able to control for employment level variables that might be associated with job crafting behaviors.

## Conclusion

Job crafting is an employee-driven, bottom-up strategy to job redesign. Our study adds to the body of evidence regarding individuals with disabilities and their crafting behaviors. In general, employees with disabilities engage in crafting behaviors less frequently than those without disabilities. Individuals factors such as education and type of disability are important determinants of crafting behaviors for persons with disabilities. As job crafting can be associated with higher levels of engagement and career growth among persons without disabilities, the findings from this research should be utilized to design programs and policies that support the career goals of people with disabilities and to maximize the benefits of employment by addressing engagement and prolonging job tenure.
