# Correction to: Job Crafting Among American Workers with Disabilities

**DOI:** 10.1007/s10926-021-09999-y

**Published:** 2021-09-13

**Authors:** Debra L. Brucker, Vidya Sundar

**Affiliations:** 1 University of New Hampshire, Institute on Disability, 10, West Edge Drive, Suite 101, Durham, NH 03824 USA; 2 Occupational Therapy Department, University of New Hampshire, 115 Hewitt Hall, 4 Library Way, Durham, NH 03824 USA

## Correction to: J Occup Rehabil (2020) 37:575–587 10.1007/s10926-020-09889-9

The article Job Crafting Among American Workers with Disabilities, written by Debra L. Brucker, was originally published electronically on the publisher’s internet portal on 13 April 2020 without open access. With the author(s)’ decision to opt for Open Choice the copyright of the article changed on 25 August 2021 to © The Author(s) 2021 and the article is forthwith distributed under a Creative Commons Attribution.

Open Access This article is licensed under a Creative Commons Attribution 4.0 International License, which permits use, sharing, adaptation, distribution and reproduction in any medium or format, as long as you give appropriate credit to the original author(s) and the source, provide a link to the Creative Commons licence, and indicate if changes were made. The images or other third party material in this article are included in the article’s Creative Commons licence, unless indicated otherwise in a credit line to the material. If material is not included in the article’s Creative Commons licence and your intended use is not permitted by statutory regulation or exceeds the permitted use, you will need to obtain permission directly from the copyright holder. To view a copy of this licence, visit http://creativecommons.org/licenses/by/4.0/.

